# Atypical dermatologic manifestations in complex regional pain syndrome: a case report

**DOI:** 10.1186/s13256-022-03466-9

**Published:** 2022-06-27

**Authors:** Brendan Langford, Thomas P. Pittelkow, Arnoley S. Abcejo

**Affiliations:** 1 Department of Anesthesiology and Perioperative Medicine, Mayo Clinic, Rochester, MN USA; 2 Department of Anesthesiology and Perioperative Medicine, Division of Pain Medicine, Mayo Clinic, Rochester, MN USA

**Keywords:** Complex regional pain syndrome, Ulcers, Multimodal analgesia

## Abstract

**Background:**

Complex regional pain syndrome is a chronic pain condition characterized by autonomic dysfunction, changes in sympathetic and vasomotor activity, and sensory and motor changes. Complex regional pain syndrome is a clinical diagnosis and may occur after trauma or surgery. Complex regional pain syndrome-related pain may occur spontaneously and is out of proportion with the inciting event. We report herein the rare case of a man who developed concomitant painful generalized ulcerations after diagnosis of complex regional pain syndrome.

**Case presentation:**

A 43-year-old Caucasian male with history of four-extremity complex regional pain syndrome type 2 secondary to right rotator cuff surgery performed at an outside hospital presented to a tertiary care center for treatment of generalized ulcerations on all extremities of unknown etiology. Dermatology performed an extensive work-up including laboratory evaluations and biopsies, which were relatively unremarkable. His ulcers were treated with vinegar-based dressings, hydrotherapy, and irrigation and debridements. He was started on methadone (replacing a home fentanyl patch), ketamine infusion, and amitriptyline in addition to his home adjuncts. He obtained good symptom control, improved sleep, and diminished cognitive slowing, compared with his fentanyl patches.

**Conclusion:**

This case report emphasizes an atypical case of generalized ulceration formation in the setting of complex regional pain syndrome. This case highlights the challenging nature of treating complex regional pain syndrome and using multimodal analgesia to target various nociceptive receptors to successfully reduce symptoms.

## Background

Complex regional pain syndrome (CRPS) is a chronic pain condition typically characterized by regionally distributed autonomic dysfunction, changes in sympathetic and vasomotor activity, and aberrations in sensation [1]. CRPS-related pain is challenging to treat as it often occurs spontaneously, is out of proportion with the inciting event with dysesthetic and allodynic features, and cannot be explained by the initial event alone [1]. CRPS may occur after trauma, cerebrovascular accident, or surgery and usually affects a single extremity [1, 2]. Unfortunately, the pathophysiology of CRPS remains elusive and likely involves dysfunction of both peripheral and central pain processing pathways and central sensitization, where an abnormal state of neuronal activation and increased nociception is generated [2]. There are often several dermatologic findings associated with this condition, with altered skin color, edema, and erythema being seen in the majority of CRPS patients [3]. Other dermatologic conditions that rarely may be seen include dermatitis, folliculitis, bullae, erythematous papules, and mild ulcerations [3]. We report herein the rare case of a man who developed severely painful generalized ulcerations 10 months after being diagnosed with CRPS.

## Case presentation

A 43-year-old Caucasian male with past medical history of hypogonadism (on testosterone injections biweekly), chronic back pain, nephrolithiasis, and CRPS type 2, secondary to shoulder surgery performed at an outside hospital due to right rotator cuff and shoulder labrum injury, presented to our emergency department for severe extremity pain with new generalized ulcerations of unknown etiology. The patient complained of diffuse body pain that initially started in the right upper extremity and had been gradually worsening over the last 1.5 years. On presentation, the pain was scored as 9 on an 11-point numeric rating scale (0 = no pain; 10 = most severe possible pain). He described the pain with dysesthetic qualities, including a stabbing sensation where the ulcers were located and a burning sensation throughout his body. In addition to the diffuse pain, his wound management program had been unsuccessful in helping his wounds heal, with constant open sores and bleeding.

Approximately 18 months prior, the patient developed an inflammatory brachial plexopathy with new-onset burning and numbness following a right shoulder operation. Initially, the concern was for Parsonage–Turner syndrome related to the postinflammatory neuropathy that may exist after surgery. However, he experienced sympathetically mediated alteration in skin coloration, hyperhidrosis, and vasomotor instability and was ultimately diagnosed with CRPS. Symptomatic treatments included both conservative (physical therapy and oral medications) and interventional techniques, including two fluoroscopic-guided stellate ganglion blocks that provided mild relief of symptoms. Over the next few months, his condition worsened, and eventually CRPS-like features including dysesthetic pain progressed to the other three extremities as well. Given the multifocal appendicular involvement, the patient received fluoroscopic-guided bilateral lumbar sympathetic blocks, with minimal improvement. Six months after his CRPS diagnosis, the patient developed large ulcerative lesions throughout all four extremities that initially began “as a patch of blisters that stretched from his dorsal wrist to inferior mid-forearm.” The patient went to a local dermatologist, who performed biopsies that were inconclusive. He was started on a trial of steroids with 80 milligrams (mg) prednisone oral daily. Despite initiation of steroids, his ulcerations continued to worsen. He started using triple antibiotic cream and taking Epsom salt baths. He had a colonoscopy that did not show any evidence of inflammatory bowel disease. He was also receiving weekly therapy by a licensed psychologist.

The patient lived at home in a healthy, supportive environment with his wife and children. He was unemployed at the time of his presentation. He denied alcohol or illicit drug use. He had 1 pack-year cigarette history; however, he quit tobacco use over 10 years before his presentation. The patient did not have any known family history of ulcerating disorders.

**Fig. 1:**
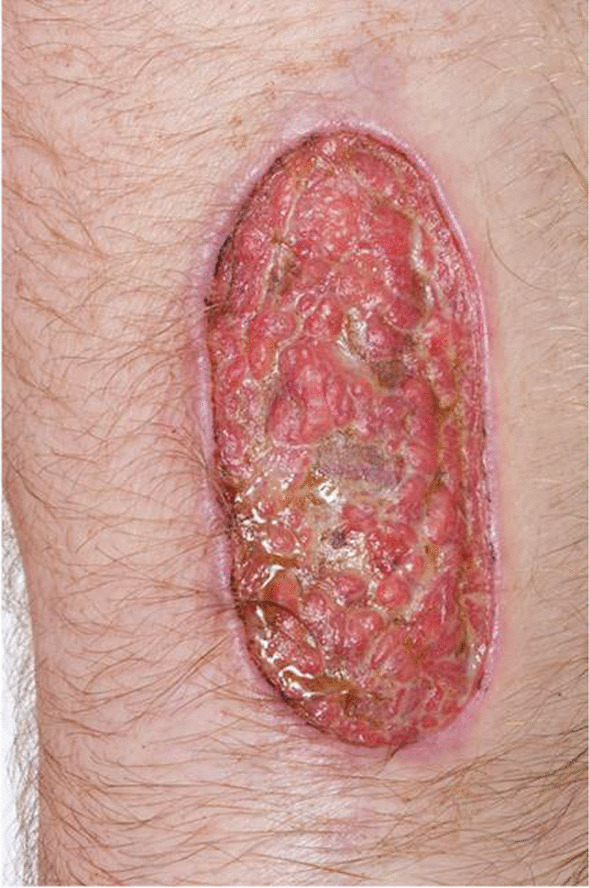
Erythematous ulcer with a well-defined non-heaped-up border on the right upper extremity

**Fig. 2:**
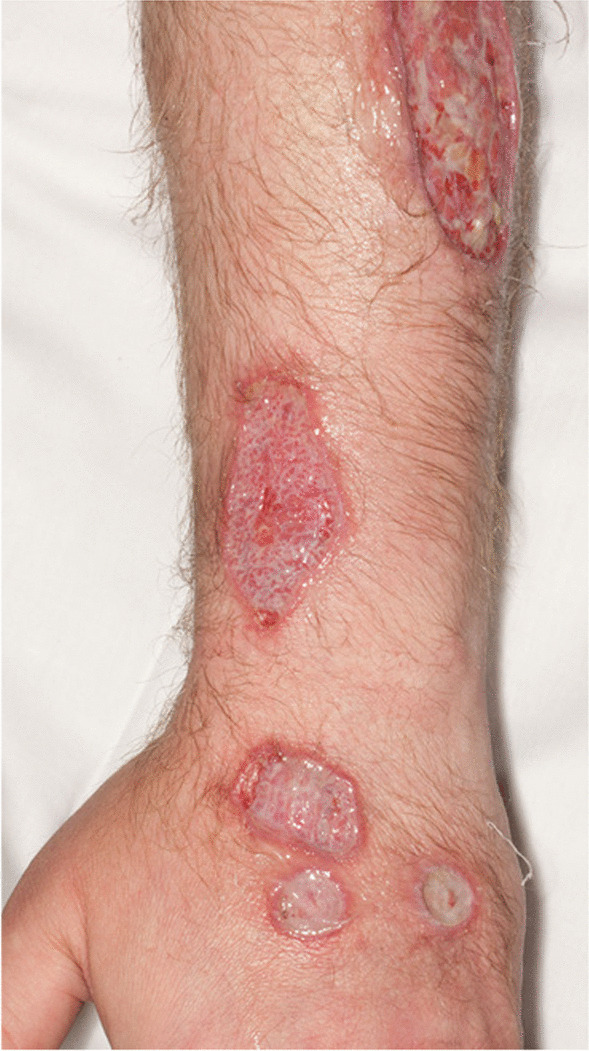
Multiple ulcers of varying severity and sizes noted on the left upper extremity

The patient’s vital signs on initial presentation included a temperature of 36.9 °C, respiratory rate of 20 breaths per minute, heart rate of 75 beats per minute, and blood pressure of 170/99 millimeters of mercury (mmHg). On physical examination, his bilateral arms had “multiple shallow-based ulcers with a necrotic center but [had] granulation tissue once some of the fibrin [was] removed.” The ulcers had a non-heaped-up, discretely demarcated erythematous border (Figs. 1, 2). There was no odor. On the bilateral lower extremities, there were large, deeper ulcerations with exposed granulation tissue. The patient did not have any ulcers on his chest, abdomen, or back. On neurologic examination, the patient was alert and oriented to person, place, and time. Cranial nerves II through XII were intact. Strength was limited by give-way weakness in the setting of his pain. He had normal deep tendon reflexes, normal sensory examination, and normal gait. The patient was admitted to the dermatology service and underwent an extensive yet unrevealing workup. Hemoglobin was 14.5 grams per deciliter, platelet count was 225,000 per microliter, and white count was 15,000 per microliter. Anti-phospholipids, anti-dsDNA, rheumatoid factor, anti-cyclic citrullinated peptide, antinuclear antibody, and antineutrophil cytoplasmic antibodies were all normal. His erythrocyte sedimentation rate and C-reactive protein were both within normal limits, as well. An amyloid study was performed but was negative. During work-up, he was found to have a small monoclonal IgM lambda within the gamma fraction. Hematology recommended obtaining immunoglobulin levels and free light chains, which were normal. Bacterial and fungal tissue cultures were obtained from the ulcers, which revealed pseudomonal and enterococcus growth. He was started on levofloxacin. Biopsies were inconclusive, showing relatively normal skin. His ulcers were treated with vinegar-based dressings, hydrotherapy with potassium permanganate, and multiple irrigation and debridements. Superficial ulcers were treated with petroleum jelly, gauze, and elastic bandages. Hyperbaric oxygen therapy was considered, but ultimately not performed due to the history and location of the lesions.

Electromyography showed an absent right medial antebrachial cutaneous sensory nerve response, with the remainder of the nerve conduction studies being normal. There was no evidence of a generalized peripheral neuropathy or of multiple mononeuropathies.

The inpatient pain medicine service was consulted immediately on presentation given his complex analgesic history. At home, the patient was on a fentanyl transdermal patch 50 micrograms per hour (mcg/hour) with intermittent use of morphine 15–30 mg by mouth every 3 hours. Adjunctive treatments included clonazepam 1 mg oral twice daily, clonidine 0.4 mg oral twice daily, gabapentin 1200 mg oral three times daily, and nabumetone 1000 mg oral twice daily. He also took escitalopram 20 mg oral daily for treatment of depression and anxiety. He endorsed both neuropathic and somatic components to his pain. Pain was exacerbated with movement and relieved with pain medications. While in the hospital, he was transitioned to methadone with increasing doses to 15 mg by mouth three times per day. He was also started on a ketamine infusion at 0.2 milligrams per kilogram per hour (mg/kg/hour), amitriptyline 50 mg oral daily, and acetaminophen 500 mg oral every 6 hours. He remained on a ketamine infusion for 5 days, with dosages ranging from 0.1 to 0.3 mg/kg/hour. Morphine was decreased from 15 to 45 mg oral every 3 hours as needed to 7.5–15 mg every 3 hours as needed. With these changes there was good symptom control, improved sleep, and diminished cognitive slowing, compared with his fentanyl patches. He also was able to change his clothes more easily. The patient’s final diagnosis was generalized ulcers in the setting of CRPS. The patient was discharged from the hospital.

The patient was seen 6 months later in our lymphedema clinic for edema in his lower extremities. At that time, he was independent with light activities but needed assistance for heavy care and activities. He was using a cane for ambulation. It was thought that, for the edema, the patient would benefit from an organized compression program. He continued to have wound care provided at a clinic closer to his home.

## Discussion

We report the rare case of a relatively healthy 43-year-old Caucasian male who developed severely painful generalized ulcerations 10 months after being diagnosed with CRPS at an outside hospital. He underwent an extensive workup that ultimately led to a diagnosis of CRPS with generalized ulcerations. His pain was managed with a multimodal analgesic approach, and his ulcers with vinegar-based dressings, hydrotherapy, and irrigation and debridements. Our case is unique in that, although mild ulceration has been reported in CRPS cases [3], we are unaware of case reports detailing severe widespread ulcerations involving all four extremities in CRPS patients. Ulcerations in this patient population are possibly secondary to skin hypoxia leading to impaired blood flow to affected limbs [4]. Initially, the ulcerations were thought to be pyoderma gangrenosum; however, serial biopsies refuted this diagnosis. Conditions that may be related to pyoderma gangrenosum such as inflammatory bowel disease were also excluded. The patient underwent an otherwise negative comprehensive workup, leading to a possible diagnosis of generalized ulcerations in the setting of CRPS.

Complex regional pain syndrome is separated into two categories. Type 1, previously known as reflex sympathetic dystrophy (RSD), most often occurs in the setting of illness or injury without direct nerve damage and is the more commonly diagnosed type of CRPS. Type 2, otherwise known as causalgia, is characterized by the presence of known nerve damage related to a specific nerve injury, often being seen after surgery or trauma and typically exhibiting more neuropathic pain symptoms including allodynia, dysesthesia, and hyperalgesia [1]. As mentioned above, CRPS is a clinical diagnosis given the often overlapping features of nociceptive and neuropathic pain symptoms. The Budapest criteria contain four diagnostic components to help aid in making a diagnosis of CRPS: sensory, vasomotor, pseudomotor/edema, and motor/trophic [5]. If a patient has all four of these diagnostic components, then the diagnosis of CRPS becomes more certain (sensitivity 0.95, specificity 0.81) [5].

The principal treatment strategy for CRPS remains optimizing functional performance while attempting to reduce life interference due to pain. Treatment options for CRPS that may be beneficial include physical therapy focused on functional use of the limb and desensitizing modalities, neuropathic oral medications (e.g., gabapentin and tricyclic antidepressants), bisphosphonates, intermittent steroids, opioids, ketamine, clonidine, peripheral sympathetic blockade (e.g., stellate ganglion blocks, lumbar sympathetic blocks, brachial plexus blocks), dorsal root ganglion (DRG) stimulation, spinal cord stimulation (SCS), and intrathecal targeted drug delivery (TDD) [6, 7]. Spinal cord stimulation was considered for this patient; however, due to his numerous open wounds, active infection concerns, and the diffuse nature of his CRPS, the pain medicine team felt that the risks outweighed the benefits. Despite multiple treatment options, many of these patients may be difficult to treat secondary to the complexity of the condition. Our patient already had multiple stellate ganglion blocks and lumbar sympathetic blocks, which were minimally helpful. He was previously on opioids and multiple adjunctive medications. With the transition to methadone and the addition of ketamine, we started to target more nociceptive pain receptors, specifically *N*-methyl-d-aspartate (NMDA) receptors, as previous literature has demonstrated the decrease of CRPS-related pain with this multimodal analgesic regimen [8]. Adopting this multimodal approach resulted in sleep improvement and reduced pain with activities of daily living. The patient was continued on methadone in the outpatient setting. Due to the complexity of the situation, he was followed closely in the outpatient setting before his transition to the wound care clinic.

## Conclusion

This case report emphasizes an atypical case of generalized ulceration formation on all four extremities in the setting of CRPS. This case also highlights the challenging nature of treating CRPS and outlines an extensive workup for dermatologic manifestations of CRPS with an emphasis on leveraging multimodal analgesia to target various nociceptive receptors to successfully reduce symptoms. Interventional approaches with SCS or DRG stimulation can be considered for patients without active infections and ideally less widespread CRPS. Recommending a comprehensive pain rehabilitation program for CRPS patients who are refractory to standard treatments or those on high-dose opioids is an appropriate approach to address underlying psychosocial, behavioral, and physical comorbidities compounding pain-related disability while focusing on restoring physical performance and improving quality of life.

## Data Availability

Not applicable.

## Abbreviations

CRPS: Complex regional pain syndrome
RSD: Reflex sympathetic dystrophy
DRG: Dorsal root ganglion
SCS: Spinal cord stimulation
TDD: Targeted drug delivery
NMDA: *N*-Methyl-d-aspartate
mmHg: Millimeters of mercury
mg: Milligrams
mg/kg/hour: Milligrams per kilogram per hour
